# No Interaction of Barrier-to-Autointegration Factor (BAF) with HIV-1 MA, Cone-Rod Homeobox (Crx) or MAN1-C in Absence of DNA

**DOI:** 10.1371/journal.pone.0025123

**Published:** 2011-09-22

**Authors:** Ying Huang, Mengli Cai, G. Marius Clore, Robert Craigie

**Affiliations:** 1 Laboratory of Molecular Biology, National Institute of Diabetes and Digestive and Kidney Diseases, National Institutes of Health, Bethesda, Maryland, United States of America; 2 Laboratory of Chemical Physics, National Institute of Diabetes and Digestive and Kidney Diseases, National Institutes of Health, Bethesda, Maryland, United States of America; Institut National de la Santé et de la Recherche Médicale, France

## Abstract

Barrier-to-autointegration factor is a cellular protein that protects retroviral DNA from autointegration. Its cellular role is not well understood, but genetic studies show that it is essential and depletion or knockout results in lethal nuclear defects. In addition to binding DNA, BAF interacts with the LEM domain, a domain shared among a family of lamin-associated polypeptides. BAF has also been reported to interact with several other viral and cellular proteins suggesting that these interactions may be functionally relevant. We find that, contrary to previous reports, BAF does not interact with HIV-1 MA, cone-rod homeobox (Crx) or MAN1-C. The reported interactions can be explained by indirect association through DNA binding and are unlikely to be biologically relevant. A mutation that causes a premature aging syndrome lies on the previously reported MAN1-C binding surface of BAF. The absence of direct binding of BAF to MAN1-C eliminates disruption of this interaction as the cause of the premature aging phenotype.

## Introduction

Barrier-to-autointegration factor (BAF/BANF1) is cellular protein that was identified as a factor that blocks autointegration of retroviral DNA [1]. The role of BAF for the host cell is not well understood, but knockdown by siRNA or genetic knockout results in a lethal phenotype that exhibits defects in nuclear morphology and division [2]–[5]. BAF is a dimer in solution and the structure of the dimer has been determined by NMR and X-ray crystallography [6], [7]. Each subunit of the dimer binds double stranded DNA non-specifically and BAF therefore bridges together double stranded DNA molecules [8]; DNA binding does not induce any conformational changes in the BAF dimer. At high DNA concentration bridging results in intermolecular aggregation. At low DNA concentration, intramolecular bridging results in compaction of DNA. BAF induced compaction of DNA molecules can be visualized by total internal reflection fluorescence microscopy (TIRFM) [9]. DNA stretched out by buffer flow condenses into a tight ball upon addition of BAF. We hypothesize that such condensation of retroviral DNA by BAF in the cytoplasm makes it refractory to autointegration. BAF also interacts with the LEM domain [10], a domain that is shared among Lamin-Associated Polypeptides, Emerin, and Man-1, proteins [11]. An NMR structure of BAF in complex with the LEM domain of Emerin reveals that that the BAF dimer binds a single LEM domain [12]. Thus BAF potentially forms complexes in which the dimer is associated with a single LEM domain protein. Since each BAF dimer binds only one LEM domain, each complex contains only one of the LEM domain proteins and each may differ in their functional properties. BAF is the primary substrate for phosphorylation by vaccinia-related kinase 1 (VRK1) [13], [14]. Vrk1 phoshorylates N-terminal residues of BAF and phosphorylated BAF no longer binds DNA. Although the interaction of BAF with DNA and the LEM domain is well understood, much remains to be learned concerning the essential role of BAF for the cell.

In addition to interacting with DNA and the LEM domain, BAF has been reported to interact with other cellular and viral proteins. These include HIV-1 matrix (MA) [15], cone-rod homeobox (Crx) [16] and the C-terminal domain of MAN-1 (MAN1-C) [17]. We sought to probe the structural basis of these interactions in order to better understand the higher order complexes that BAF may form in the cell. Contrary to the previous reports, we found that none of the above proteins interact with BAF. The reported interactions are likely mediated through DNA binding. Since any two DNA binding proteins can indirectly interact through DNA, we conclude that evidence does not support functionally relevant interaction between BAF and MA, Crx, or MAN1-C.

## Results and Discussion

**Figure 1:**
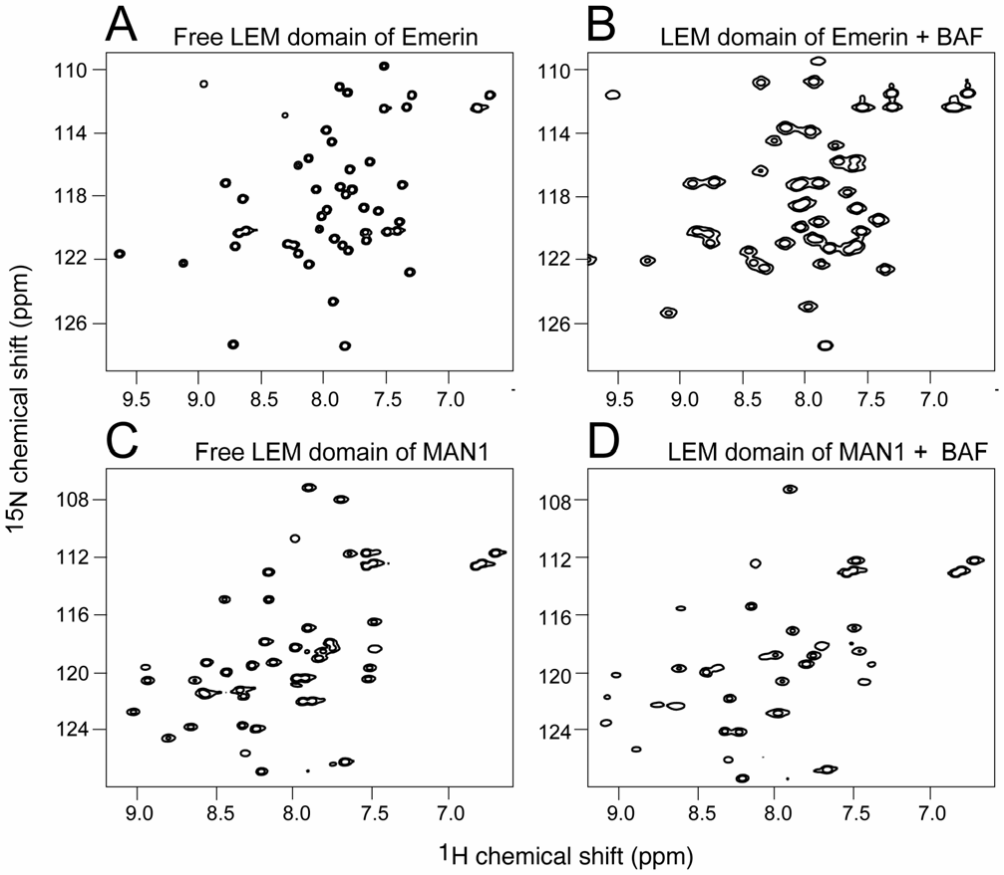
^1^H-^15^N HSQC spectrum of the Emerin (panel A and B) and MAN1 (panel C and D) LEM domains in the absence and presence of BAF. Spectra were collected on 50 µM ^15^N-labeled Emerin LEM domain (panel A) or 50 µM ^15^N-labeled Emerin LEM domain plus 200 µM BAF_2_ (panel B). Panels C and D show the results of the same experiment substituting 50 µM MAN1 LEM domain for the Emerin LEM domain.

We used 2D ^1^H-^15^N heteronuclear single quantum correlation (HSQC) spectroscopy to monitor interactions between BAF and its putative binding partners by NMR. In general, each cross-peak in the ^1^H-^15^N HSQC spectrum corresponds to one amino acid in the ^15^N-labeled protein. Changes in local environment caused by altered conformation or interaction with other proteins shift or alter the intensity of related cross-peaks. The HSQC spectrum therefore represents a fingerprint of the system and can be used to map binding interfaces. Figure 1 shows the changes in the ^1^H-^15^N HSQC spectrum of LEM domains upon interaction with BAF. Figure 1A shows the ^1^H-^15^N HSQC spectrum of the LEM domain of Emerin. Upon incubation with BAF there are major changes in many of the peaks corresponding to residues that interact with BAF (Figure 1B). Comparison of the ^1^H-^15^N HSQC spectrum of the ^15^N-labeled LEM domain of MAN1 (Figure 1C) with the same domain bound to BAF (Figure 1D) also reveals considerable differences.

**Figure 2:**
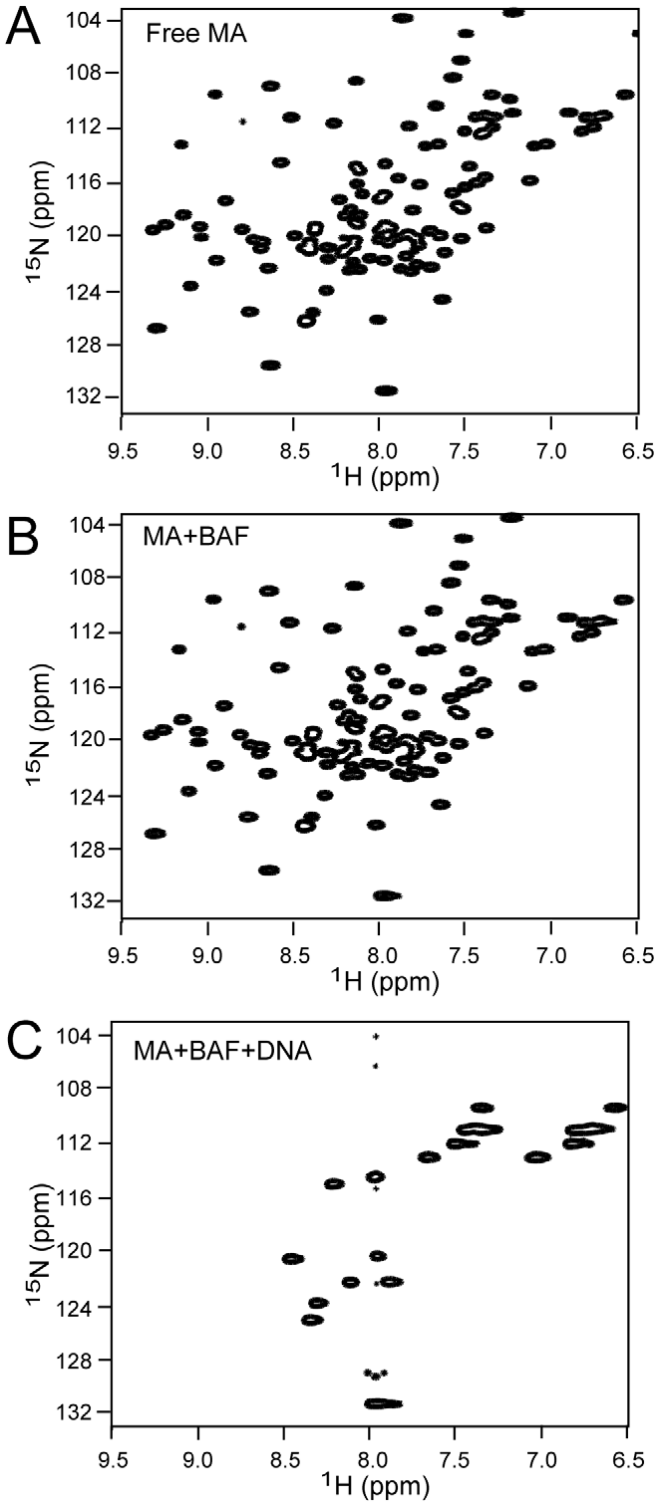
^1^H-^15^N HSQC spectrum of: (A) 0.5 mM ^15^N-labeled free HIV-1 MA, (B) 0.5 mM ^15^N-labeled MA plus 1 mM unlabeled BAF, (C) 0.5 mM ^15^N-labeled MA plus 1 mM unlabeled BAF and 2 mM 16 mer DNA.

We initially set out to probe the interaction surfaces of BAF and HIV-1 MA by NMR. Figure 2A shows the ^1^H-^15^N HSQC spectrum of ^15^N-labeled MA. To our surprise, addition of BAF to the MA sample did not result in any changes in the spectrum even at the protein concentrations used for the NMR measurements. We conclude that BAF and MA do not directly interact. Then how might the previously reported interactions [15] be explained? BAF and MA both bind DNA and we propose the protein preparations contained sufficient DNA to allow an apparent interaction between BAF and MA through DNA binding. Consistent with this interpretation, the low micromolar range reported apparent affinity of MA for BAF is similar to the affinity of MA for DNA [18]. BAF binds DNA more tightly and DNA condensation can present a kinetic barrier to dissociation [9]. In our hands, both BAF and MA tend to co-purify with DNA and extensive washing of columns at high ionic strength is required to remove all traces of DNA during purification. Some of the previously reported experiments [15], [17] were also carried out with BAF synthesized in an *in vitro* coupled transcription/translation system and this protein will have contained carried over template plasmid DNA. As expected, when DNA is added to the mixture of BAF and MA the spectrum radically changes (Figure 2C). We note that the number of cross-peaks is greatly diminished in contrast with addition of DNA to MA, alone which shifts and broadens a subset of peaks without reducing their number [19]. The DNA used for this experiment was a 16 mer duplex oligonucleotide which demonstrates that BAF and MA can simultaneously bind to very short DNAs.

**Figure 3:**
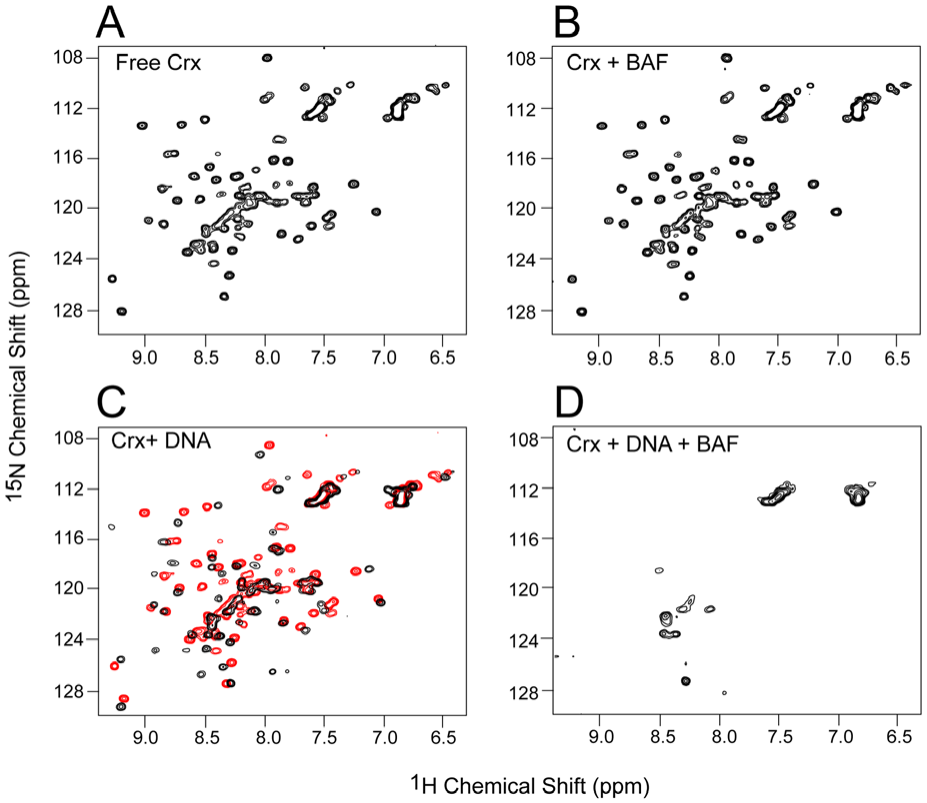
^-1^H-^15^N HSQC spectra of Crx homeodomain. (A) 30 µM free ^15^N-labeled Crx. (B) 30 µM free ^15^N-labeled Crx plus 200 µM BAF_2_. (C) 30 µM free ^15^N-labeled Crx plus 30 µM 16 mer DNA (black). The spectrum in the absence of DNA is superimposed in red. (D) 30 µM free ^15^N-labeled Crx plus 200 µM BAF_2_ and 30 mM 16 mer DNA.

Many of the proteins reported to interact with BAF are DNA binding proteins. We therefore decided to reexamine their binding in light of our experience with HIV-1 MA. Cone-rod homeobox (Crx) protein is a transcription factor that was identified as a BAF interacting protein in a yeast two-hybrid screen [16]. Co-immunopreciptation and pull-down assays supported the conclusion that BAF and Crx interact directly. Figure 3A and 3B show the ^1^H-^15^N HSQC spectrum of ^15^N labeled Crx in the absence and presence of BAF, respectively. The spectra are identical, unambiguously indicating that BAF and Crx do not interact directly. Addition of a 16 mer duplex DNA to the Crx results in a shift of a subset of the cross-peaks as expected for binding of DNA by Crx (Figure 3C, compare the spectrum in the presence of DNA (black) with the superimposed spectrum in the absence of DNA (red)). Addition of both DNA and BAF to Crx results in disappearance of most of the cross-peaks (Figure 3D), indicative of the formation of large complexes of Crx, BAF and DNA.

**Figure 4:**
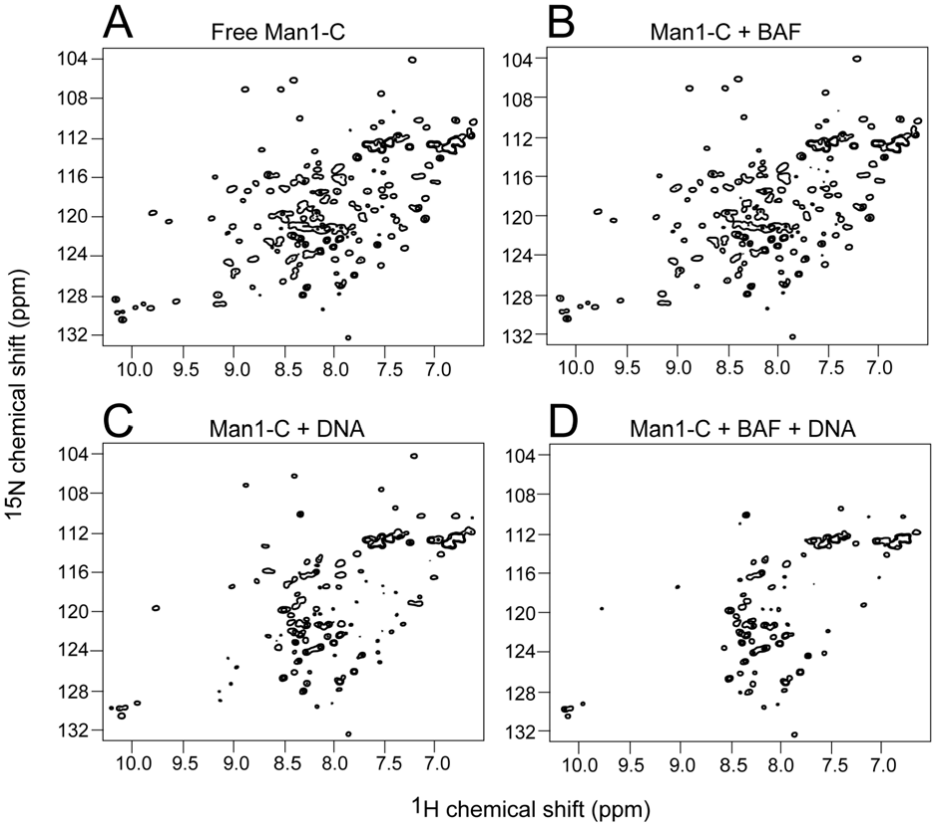
^1^H-^15^N HSQC spectra of MAN1-C. (A) 50 µM free ^15^N-labeled MAN1-C. (B) 50 µM free ^15^N-labeled MAN1-C plus 200 µM BAF_2_. (C) 50 µM free ^15^N-labeled MAN1-C plus 50 µM 16 mer DNA. (D) 50 µM free ^15^N-labeled MAN1-C plus 200 µM BAF_2_ and 50 µM 16 mer DNA.

MAN1 is an inner nuclear membrane protein that contains a LEM domain near its N-terminus that binds BAF. The C-terminal domain of MAN1 (MAN1-C) has been reported to independently bind BAF [17] in addition to the transcription factors GCL and Btf. We labeled MAN1-C with ^15^N and recorded the ^1^H-^15^N HSQC spectrum (Figure 4A). Addition of BAF to the MAN1-C resulted in no significant change in the spectrum (Figure 4B) demonstrating that these proteins do not interact directly. Interestingly we find that MAN1-C binds DNA (Figure 4C). Addition of DNA and BAF to MAN1-C results in additional disappearance and shifts in peaks demonstrating that BAF and MAN1-C form a large complex with DNA, although BAF and MAN1-C do not interact directly.

**Figure 5:**
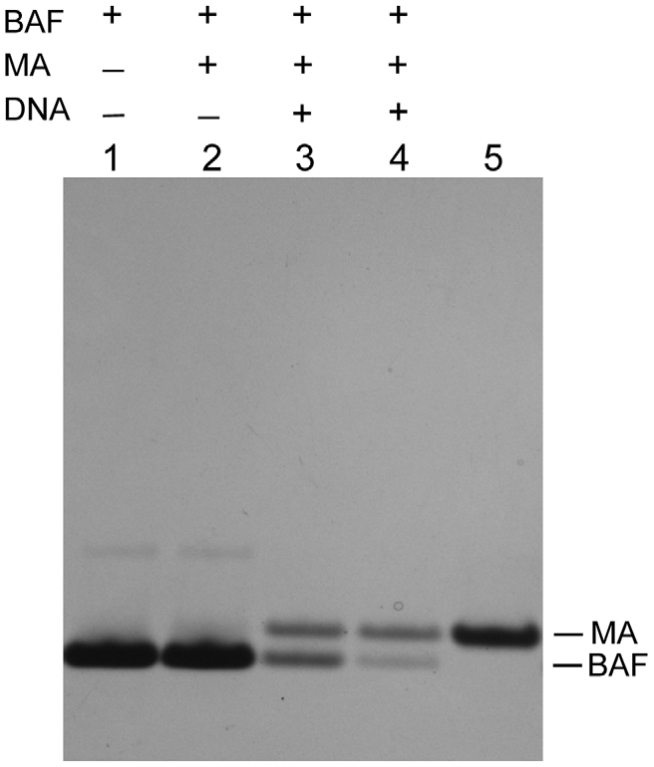
Co-elution of MA with BAF in pull-down assays in the presence of DNA. Pull-down assays were performed on a Ni chelating sepharose column equilibrated with binding buffer. BAF was then applied in binding buffer and the column was extensively washed. Sonicated salmon sperm DNA (50 µl 0.02 (lane 3) or 0.06 µg/µl (lane 4)) was then applied and the washing step was repeated. 50 µl of 0.15 µg/µl MA was then applied in binding buffer and the washing step was repeated. Finally, BAF was eluted with 70 µl 1 M imidazole. Proteins were electrophoresed in a 4–12% Bis Tris NuPAGE gel (Invitrogen) and stained with Coomassie. Lane 5 shows MA alone as a mobility standard. In the presence of DNA some BAF remains trapped on the column during the elution step because it forms a cross-bridged network with DNA; it elutes with SDS (data not shown).

To confirm that DNA contamination can confound the interpretation of pull-down assays for protein-protein interactions we carried out such an assay for BAF and MA interaction in the absence and presence of DNA. His-tagged BAF was bound to a Ni chelating sepharose column. MA was then added to the column in the absence or presence of DNA. After extensive washing BAF was eluted with imidazole. In the absence of DNA only BAF eluted from the column (Figure 5, lane 2). However, in the presence of DNA, MA co-eluted with the BAF (lanes 3 and 4).

The mutation of Ala12Thr in BAF has been identified as the cause of a human Hereditary Progeroid Syndrome [20]. Ala12 is surface exposed but does not map to either the DNA or LEM domain binding surfaces of BAF, so the effects of this mutation are unlikely to involve disruption of DNA or LEM domain binding. It was proposed that the mutation might affect the interaction of BAF with other proteins, its subcellular localization or stability [20]. Indeed Ala12 lies on the surface of BAF that was implicated in binding MAN1-C [17] and disruption of the MAN1-C/BAF interaction would have been a reasonable candidate for the primary effect of the mutation. Our finding that MAN1-C does not interact with BAF eliminates this model. Although we cannot ignore the possibility that Ala12Thr disrupts an interaction with a factor yet to be identified, the reduced abundance of BAF in the mutant cells [20] suggests a primary effect on protein stability.

Pull-down assays and co-immunoprecipitation are commonly used to screen for protein-protein interactions because of their simplicity and convenience. However putative interactions identified by such assays need to be confirmed and substantiated by more direct biochemical and biophysical methods. We conclude that, contrary to previous reports, BAF does not interact with HIV-1 MA, Crx, or MAN1-C.

## Materials and Methods

### Protein Expression and Purification

Human BAF and the LEM domains from MAN1 (residues 1–52) and Emerin (residues 1–47) were cloned and purified as described [12]. MA was purified as described [19].

MAN1-C (residue 650–911) was subcloned into a modified pET-32a vector [12] to form a thioredoxin fusion protein with a His_6_ tag and expressed in Escherichia coli strain BL21(DE3) (Novagen). The construct was verified by DNA sequencing. *E. coli* transformed with the MAN1-C vector were grown in minimal medium with ^15^NH_4_Cl and glucose as the nitrogen and carbon sources, respectively. Cells were induced with 1 mM isopropyl D-thiogalactopyranoside at A_600_ 1.0, and harvested by centrifugation 3 h following induction. After harvesting, the cell pellet was resuspended in 50 ml (per liter of culture) of 50 mM Tris, pH 7.4, 1 M NaCl, 10 mM imidazole, and 1 mM phenylmethylsulfonyl fluoride. The suspension was lysed by two passages through a microfluidizer and centrifuged at 10,000× g for 40 min. The supernatant fraction was loaded onto a HisTrap HP column (5 ml per 2 liters of culture; GE Healthcare) equilibrated with 1 M NaCl, 10 mM imidazole, 20 mM Hepes pH 7.5, 10% glycerol, 2 mM 2-mercaptoethanol, and the column was extensively washed with equilibration buffer. The fusion protein was eluted with a 100 ml gradient of imidazole (25–500 mM) in the same buffer. The protein was then dialyzed against 20 mM Tris pH 7.5, 200 mM NaCl, and digested with thrombin (10 NIH units/mg of protein) for 2 hr at room temperature. Thrombin was then removed by passage over a benzamidine sepharose column. The cleaved His_6_-thioredoxin was removed by loading the digested proteins over a HisTrap HP column. MAN1-C was further purified by gel filtration on Sephadex-75 gel filtration column (GE Healthcare) equilibrated with 25 mM potassium phosphate pH 6.5, 150 mM NaCl, 2 mM 2-mercaptoethanol.

pGEX-4T-2 GST expression vector encoding the Crx homeodomain (residues 34–107) plus five N-terminal and nine C-terminal flanking amino acid residues was a gift from Dr. Shiming Chen (Washington University in St. Louis), and was expressed in the *E. coli* strain BL21(DE3) the same way as described for MAN1-C above. Cells were lysed the same way as described for MAN1-C above except that the buffer was 50 mM Tris pH 7.4 containing 0.5 M NaCl. Before eluting the GST fusion protein from the glutathione Sepharose 4B, the column was washed with at least 20 column volumes phosphate buffered saline (PBS) plus 0.5 M NaCl until no DNA was present in the eluate. Protein fractions eluted with 50 mM Tris pH 8 containing 10 mM reduced glutathione were pooled together and dialyzed against 4L 50 mM Tris pH 7.5 containing 0.5 M NaCl and 2 mM 2-mercaptoethanol. The GST fusion tag was removed by digestion with thrombin and the Crx homeodomain was separated by gel filtration on a Sephadex-75 gel column equilibrated with 25 mM potassium phosphate pH 6.5 containing 150 mM NaCl and 2 mM 2-mercaptoethanol.

Protein samples for NMR contained 25 mM potassium phosphate pH 6.5, 2 mM 2-mercaptoethanol in 95% H_2_O and 5% D_2_O with different salt concentrations. Salt concentrations were 200 mM NaCl for free LEM domains, LEM domain complexes, Crx, Crx/BAF and Crx/BAF/DNA complexes. The salt concentration was 150 mM NaCl for MAN1-C, MAN1-C/BAF and MAN-1C-/BAF/DNA complexes. DNA used for all NMR experiments was a 16 mer DNA duplex (5′ CCAGCACAAACACCTG and its complement).

### NMR Spectroscopy

^1^H-^15^N HSQC spectra were recorded at 27°C on Bruker DRX500 and DRX600 spectrometers equipped with triple resonance Z gradient cryoprobes. Spectra were processed using the program NMRPIPE [21], and analyzed using the program PIPP [22]. For the LEM domains of Emerin and MAN-1, ^1^H-^15^N HSQC spectra were collected on samples of 50 µM free ^15^N labeled LEM domain and 50 µM LEM domains plus either 100 µM (50 µM in dimer form) unlabeled BAF or 400 µM unlabeled BAF. For MAN1-C, spectra were collected on samples of 50 µM free ^15^N labeled MAN1-C, 50 µM ^15^N labeled MAN1-C plus 50 µM BAF_2_ or 200 µM BAF_2_ and 50 µM ^15^N labeled MAN1-C plus 50 µM unlabeled BAF_2_ and 50 µM 16 mer DNA. For Crx, spectra were collected on samples of 30 µM ^15^N labeled Crx, 30 µM Crx plus 30 µM or 200 µM unlabeled BAF_2_ and 30 µM ^15^N labeled Crx plus 30 µM BAF_2_ and 30 µM 16 mer DNA.

### His-tag BAF pull-down assay

Pull-down assays were performed on a 150 µl Ni chelating sepharose columns equilibrated with 50 mM NaCl, 20 mM Hepes pH 7.5, 20 mM imidazole, 2 mM 2-mercaptoethanol (binding buffer). 50 µl of 0.3 µg/µl BAF was then applied in binding buffer. The column was then washed three times, each with 200 µl of binding buffer. 50 µl sonicated salmon sperm DNA (0.02 or 0.06 µg/µl) was then applied (when indicated) and the washing step was repeated. 50 µl of 0.15 µg/µl MA was then applied in binding buffer and the washing step was repeated. Finally, BAF was eluted with 70 µl 1 M imidazole pH 7.5. Proteins were electrophoresed in a 4–12% Bis Tris NuPAGE gel (Invitrogen) and stained with Coomassie.
